# Utilizing Dietary Micronutrient Ratios in Nutritional Research May be More Informative than Focusing on Single Nutrients

**DOI:** 10.3390/nu10010107

**Published:** 2018-01-19

**Authors:** Owen J. Kelly, Jennifer C. Gilman, Jasminka Z. Ilich

**Affiliations:** 1 Abbott Nutrition, 2900 Easton Square Place, 01C13A/ES1, Columbus, OH 43219, USA; jennifer.gilman@abbott.com; 2 Department of Nutrition, Food and Exercise Sciences, Florida State University, Tallahassee, FL 32306, USA; jilichernst@fsu.edu

**Keywords:** dietary guidelines, micronutrient ratios, dietary patterns, micronutrients, dietary reference intakes, daily values, mineral, vitamin

## Abstract

The 2015 US dietary guidelines advise the importance of good dietary patterns for health, which includes all nutrients. Micronutrients are rarely, if ever, consumed separately, they are not tissue specific in their actions and at the molecular level they are multitaskers. Metabolism functions within a seemingly random cellular milieu however ratios are important, for example, the ratio of adenosine triphosphate to adenosine monophosphate, or oxidized to reduced glutathione. Health status is determined by simple ratios, such as the waist hip ratio, or ratio of fat mass to lean mass. Some nutrient ratios exist and remain controversial such as the omega-6/omega-3 fatty acid ratio and the sodium/potassium ratio. Therefore, examining ratios of micronutrients may convey more information about how diet and health outcomes are related. Summarized micronutrient intake data, from food only, from the National Health and Nutrition Examination Survey, were used to generate initial ratios. Overall, in this preliminary analysis dietary ratios of micronutrients showed some differences between intakes and recommendations. Principles outlined here could be used in nutritional epidemiology and in basic nutritional research, rather than focusing on individual nutrient intakes. This paper presents the concept of micronutrient ratios to encourage change in the way nutrients are regarded.

## 1. Introduction

The basic science is not physics or mathematics but biology—the study of life. We must learn to think both logically and bio-logically.

—Edward Abbey [1]

The foundation of nutritional science is rooted in singling out specific foods, nutrients or other compounds, such as phytochemicals; historically protein was once considered the only nutrient (we recommend reading A Short History of Nutritional Science: Part 1 [2], 2 [3], 3 [4] and 4 [5]). This very early search for foods/nutrients that would cure diseases (predominately acute conditions) remains the principle of nutritional science today, in the setting of our modern sedentary lifestyle and even in the face of global obesity, diabetes and other chronic disease. Current nutritional issues are very different from sailors receiving fresh fruit and vegetables to alleviate scurvy caused by vitamin C deficiency; the current nutritional issues have a complex etiology and this is our focus. However, it is important to state that a lot has been learned from human studies with single nutrients; especially as a means to treat the nutrient deficiencies in many parts of the world [6].

The release of the 2015 Dietary Guidelines for Americans (2015 DG) has not been without controversy [7]. However, the 2015 DG focus on the whole diet (or dietary patterns), as opposed to focusing on single nutrients, as is the case with Dietary Reference Intakes (DRI). The concept of small effects from diet over time is also present in the 2015 DG, showing that it takes time for dietary/lifestyle changes to produce beneficial effects, which may be more appropriate for real life situations. Nevertheless, this is not a sudden change since the first dietary guidelines in 1980; there has been a slow shift towards dietary patterns and away from single nutrients [8]. Some food patterns have a higher inherent concentration of beneficial nutrients [9], as well as a potentially better plant to animal energy subsistence ratio, compared to modern diets [10].

Future dietary guidelines could also consider the burden of chronic disease and prescription use in the US population. Zhong et al. estimate approximately 70% of Americans are prescribed at least one drug; and over 50% are prescribed two drugs [11]. Current guidelines focus on the “healthy” population. “Healthy” may not be easy to define [12], however an appropriate and applicable definition is required to aid future guidelines. “Healthy” may merely be the absence of a condition/disease, or it is possible that “healthy” can coexist with a chronic condition/disease, for example metabolically healthy obese [13]. This suggests a certain metabolic state may help to define “healthy”.

Regardless, the dietary pattern approach should consider the role of all nutrients in combination within human metabolism, advising that combinations (ratios) of nutrients in a complete diet, are more informative than each single nutrient’s contribution. All nutrients are required to allow metabolism to operate normally and there may be optimal physiological combinations (ratios) of nutrients. Thus, the dietary patterns implications are that the entire diet is more important than the sum of its parts (nutrients). Traditionally, it has not been common to present the intake values for all vitamins or all minerals relative to each other and there are no actual recommendations for a person’s total daily intake of vitamins or minerals. The following suppositions are the basis for our nutrient ratio hypothesis:

1. no person consumes a single nutrient (macro- or micro-nutrient) separately in a meal;
2. in an ideal varied diet, the nutrient intake would be different for each eating occasion;
3. nutrients do not target one particular tissue when consumed;
4. at the molecular level, nutrients are multitaskers; each nutrient does not act in one specific metabolic pathway, or alone.

While nutrient ratios are not a novel idea, this is the first time to suggest that modern nutritional science adapt these principles. This would be a very different way to think about nutrient intakes but it may bridge the gap between single nutrient based information and dietary patterns. It goes beyond the classical approach of evaluating each micro- or macro-nutrient by itself; which may be a relic of the past when deficiency type studies dominated, or it may be due to the adoption of the (drug based) randomized clinical trial model in nutrition. Regardless, nutritional science must develop new standardized models to account for the complexity of human nutrient metabolism and the lack of a true placebo to food/nutrients. Therefore, considering micronutrients in relation to one another is physiological and it is the basis for putting forward our hypothesis: micronutrient ratios may confer more comprehensive information than the concentration of a single nutrient. In this paper, we present the rudimentary foundations of this concept.

### 1.1. The Matter of Ratios

Human metabolism functions on ratios of interacting signaling molecules, enzymes and substrates. This can be as simple as the ratio of adenosine triphosphate (ATP) to adenosine monophosphate (AMP), or the ratio of oxidized to reduced glutathione. At the whole body level, simple ratios such as the waist hip ratio, ratio of fat mass to lean mass, or even upper to lower body fat mass [14], the ratio of sex hormones [15,16], the ratio of electrolytes [17], or the glucose to insulin ratio [18] provide more information than the individual components. The Lee Index also known as the Nutritive Ratio (the cube root of body weight divided by the naso-anal length) was a means to measure obesity [19]. Prostaglandin E_2_ (PGE_2_) is known to be biphasic [20]; at lower concentrations, it is less inflammatory than at higher concentrations. In both cases, PGE_2_ is still present in the milieu suggesting the ratio of PGE_2_ to other molecules may have the effect. A new measure; the Dietary Inflammatory Index (DII) was developed by Hébert and colleagues [21], an update from the original Inflammatory Index based on C-reactive protein’s response to dietary components [22], to include more inflammatory measures (six in total: interleukins 1β, 4, 6 & 10, tumor necrosis factor alpha and C-reactive protein) and expand to a population based index. This DII effectively quantifies the ratio of inflammatory foods to anti-inflammatory foods. The DII correlates with other well-known measures of diet quality; the Healthy Eating Index-2010 (HEI-2010), the Alternative Healthy Eating Index (AHEI) and the Dietary Approaches to Stop Hypertension Index (DASH) [23]. The DII is gaining interest and a higher DII score (more inflammatory) has been associated with insulin resistance [24], risk of first myocardial infarction [25] circulatory conditions [26] and colorectal cancer risk [27]. These data show that different diets and ratios of dietary components and thus different levels of nutrients and their ratios may be an important consideration in chronic disease.

Certain simple nutrient ratios already exist; for example; the sodium/potassium ratio [28], calcium/magnesium ratio [29], omega-6 (n-6) to omega-3 (n-3) polyunsaturated fatty acids ratio [30] and the eicosapentaenoic acid/arachidonic acid ratio [31] (the usefulness of these latter ratios remains controversial as there may be a threshold effect of n-3 fatty acids [32]). Within human metabolism there may also be different ratios of micronutrients in each tissue/organ because the metabolic configurations differ in each organ [33], as blood and other easy to measure endpoints are the focus of most research. The status of micronutrients within different tissues/cells represents a large research gap for nutritional science in general. In human metabolism, it is rare to have a biological on and off switch, instead ratios and proportions of synergistic and opposing molecules (including nutrients) determine outcomes; the same may occur for micronutrients.

Other ratios are also important in human nutrition; these are the ratios of certain minerals that inhibit the absorption of each other. An excellent early paper outlines the mineral interactions relevant to nutrition between the macroelements, as well as interactions among the microelements [34]. At its most basic, minerals with similar physiochemical properties will be antagonists. The most important mineral ratios in relation to human nutrition then and now seem to be sodium-potassium and iron-zinc-copper interactions. The complexity of mineral interactions is not limited to just the minerals alone, other food components effect absorption, e.g., vitamin C enhances iron absorption and protein enhances zinc absorption. This suggests that nature may have already built in measures to mitigate mineral interactions within a whole food diet and certain dietary patterns may be better than others for maximizing mineral absorption. Conversely, large doses of single minerals may result in negative mineral interactions as they are not accompanied by the other food components inherent in the standard food source of the mineral (e.g., potassium from bananas and zinc from seafood). Mineral interactions, mineral–vitamin interactions (e.g., vit D regulates blood P and Ca) [35] and gene–nutrient interactions are remarkable and highlight the complexity of nutritional science. All these interactions occur after ingesting food but they may be ultimately determined by the ratios of dietary micronutrients.

### 1.2. Micronutrient Ratios

We recently described how the US diet (a proxy for the Western diet) is chronically insufficient in calcium (Ca), magnesium (Mg) and potassium (K) and the fat-soluble vitamins (A, D and E) but higher in phosphorus (P) and sodium (Na) and many of the B vitamins, as assessed from the National Health and Nutrition Examination Survey (NHANES) data, from 2001 to 2012, in all age groups [36]. Therefore, vitamin insufficiencies and excesses coexist, suggesting it may be the pattern, or ratio, of micronutrients that is the key. This suggests the underlying issue is a disruption in the ratios of micronutrients in the Western diet.

It is possible that studies focused on one mineral or vitamin, while discounting the nutrient-nutrient interactions and multitasking nature of nutrients, may be one of the reasons results from nutritional trials are so varied. This nutrient ratio concept also proposes there may be an ideal daily ratio of micronutrients to maintain health and prevent chronic disease, which would be ultimately be related to certain dietary patterns, as opposed to individual DRIs.

Because each micronutrient (with a few exceptions for the trace elements) has a DRI, a daily total micronutrient recommendation exists. In other words, if an individual achieved the DRI for each micronutrient he/she would have consumed a specific quantity of minerals and vitamins and they would be in a specific ratio.

The idea of nutrient ratios is well known and many other nutritional scientists are aware that absolute vales of single or multiple nutrients may mask the bigger picture. However, this is the first paper, to our knowledge, that proposes that the dietary micronutrient ratios should be routinely reported and that a concerted effort should be sought to develop the framework and mathematics to allow robust correlations to health and disease outcomes. Here, we develop the first micronutrient ratios from the DRIs and NHANES data for the US population and show that the male and female dietary micronutrient ratios from food (not supplements) do not match those inherent in the DRIs.

## 2. Materials and Methods

For this preliminary exploration of dietary micronutrient ratios, the summarized NHANES 2001–2014 data, from food only, from What We Eat in America [37] were transposed to Microsoft Excel. The rational for using food only data was that it would provide a better benchmark for dietary intake. In addition, the most common benchmarks for diet quality, such as the HEI, are based on food only. Supplements seem to be important in modern diets to fill nutritional gaps but there is no way to measure the quality of a diet that includes supplements; this is something to add in future diet quality instruments. Total mean daily intakes as well as intakes at different eating occasions, from food only, were utilized. Ratios for minerals and vitamins (separately and combined) were calculated, with the DRI ratios used as the benchmark. Mineral and vitamin intakes, DRIs and Daily Values (DVs; a value set by the Food and Drug Administration for food labelling purposes) were converted to the milligram. Total mineral or vitamin intakes were calculated by adding together all intake levels of minerals or vitamins, or minerals and vitamins combined. While Estimated Average Requirements are typically used in population-based studies, we deliberately used the entire set of DRIs so we could establish a recommended intake value for each micronutrient, to allow ratios to be calculated in this exercise.

To calculate ratios, the intake value for a mineral or vitamin was divided by the sum of all intake values for minerals or vitamins in the set; for example, if there were three minerals:

- Ratio of mineral 1 = mineral 1/(mineral 1 + mineral 2 + mineral 3)
- Ratio of mineral 2 = mineral 2/(mineral 1 + mineral 2 + mineral 3)
- Ratio of mineral 3 = mineral 3/(mineral 1 + mineral 2 + mineral 3)

To check the calculations were correct the sum of all ratios would be = 1 (ratio of mineral 1 + ratio of mineral 2 + ratio of mineral 3 = 1).

While the ratios are important values and could be used in calculations and analysis, they are just another set of numbers. Therefore, we wanted to include an alternative way to express them, to help conceptualize ratios and have whole numbers for all micronutrients. We decided to express the intakes of micronutrients relative to one micronutrient; the simplest way was to use the lowest value to set at one. To calculate the ratio relative to a specific mineral or vitamin, the intake value for each mineral or vitamin was multiplied by the reciprocal of the reference mineral or vitamin intake value, for example if there were three minerals and mineral 3 was the lowest value, first the ratios need to be calculated:

- Ratio of mineral 1 = mineral 1/(mineral 1 + mineral 2 + mineral 3)
- Ratio of mineral 2 = mineral 2/(mineral 1 + mineral 2 + mineral 3)
- Ratio of mineral 3 = mineral 3/(mineral 1 + mineral 2 + mineral 3)

Next all ratios are multiplied by the reciprocal of mineral 3 (the lowest value):

- Ratio of mineral 1 × (1/Ratio of mineral 3)
- Ratio of mineral 2 × (1/Ratio of mineral 3)
- Ratio of mineral 3 × (1/Ratio of mineral 3) = 1

The important distinction is that ratio values represent all micronutrients but the relative ratio is only in reference to one micronutrient. For this paper, the micronutrient that is set at one means it is at a concentration of 1 mg, which puts many of the micronutrients at much higher than physiological concentrations. However, it is purely for illustrative purposes and helps support the concept that micronutrient levels are relative.

The same method was applied for the minerals and vitamins combined. To limit the size of the data presented in this manuscript and to simplify the presentation of preliminary results, the male and female 50–59-year-old age groups from the 2007–2008 and 2013–2014 NHANES surveys were used as the examples throughout the text. The same equations were used to calculate totals and ratios for the DRIs and DVs.

What We Eat in America summary tables [37] only contain dietary nutrient intake data, from food (not including supplements), for calcium (Ca), phosphorus (P), magnesium (Mg), iron (Fe), zinc (Zn), copper (Cu), selenium (Se), potassium (K) and sodium (Na). Intake data are not provided for chloride, chromium, iodine, manganese, molybdenum, fluoride, boron, nickel, silicon, vanadium and cobalt. For the vitamins, What We Eat in America does not report biotin (B_7_) and pantothenic acid (B_5_), however, vitamin A (A or retinol activity equivalents (RAE)), thiamin (B_1_), riboflavin (B_2_), niacin (B_3_), pyridoxine (B_6_), cobalamin (B_12_), folate (as dietary folate equivalent (DFE)), vitamin C (C or Vit C; as ascorbate), vitamin D (D or Vit D; as cholecalciferol and ergocalciferol), vitamin E (E or Vit E; as α-tocopherol) and vitamin K (Vit K; as menaquinone and phylloquinone) were included. Vitamin D was not included in the 2001–2002 and 2003–2004 What We Eat In America data summaries. Although these missing minerals and vitamins are associated with normal human homeostasis, they could be included in a future larger analysis of micronutrient ratios.

## 3. Results

### 3.1. Mineral Ratios

**Figure 1:**
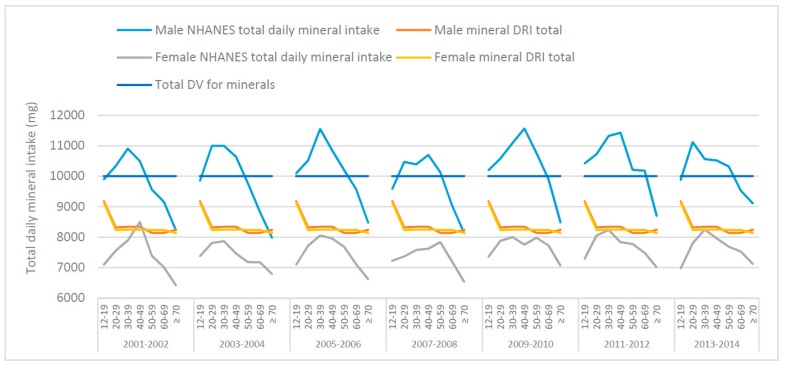
Total mean daily mineral intakes from food across various age groups, derived from National Health and Nutrition Examination Survey (NHANES) 2001–2014 data, the total Dietary Reference Intakes (DRI) and the total Daily Value (DV) for males and females.

The total mean male and female daily mineral intakes (mg), from food, from NHANES data (50–59-year-old age groups from the 2007–2008 and 2013–2014 NHANES surveys) were calculated and graphed with the totals for DRIs and DVs, as shown in Figure 1. The total mineral intake inherent in the DRIs are similar for males and females except for adjustments to Ca, Mg and Zn. With a few exceptions, NHANES data (across survey years and age groups) indicates males are consuming above, whereas females are consuming below, the total daily-recommended mineral intake derived from the DRIs. This suggests that, overall; the total daily mineral intakes from food may be too high in males but too low in females.

The ratios of minerals for the DRIs, DV and NHANES data are shown in Table 1. According to the current DRIs, males and females aged 51–70 years should consume approx. 8140 mg and 8237 mg of K, Na, Ca, P, Mg, Zn, Fe, Cu and Se per day, respectively (Table 1). The DV total for these minerals is 10,000 mg. However, NHANES data from 2007–2008 showed that the total daily mineral intake from food for males was approx. 10,131 mg and 7838 mg for females. Total daily mineral intake for NHANES 2013–2014 showed that males consumed approx. 10,178 mg while females consumed 7590 mg of K, Na, Ca, P, Mg, Zn, Fe, Cu and Se, from food (Table 1).

Generally, the total daily mineral intake from food is sufficient (per DRI) for males but not females, aged 50–59 years and does seem to decline with age (Table 1). From 2001–2006 those ages 50 or older did not meet the total DV, for 2007–2010 and 2013–2014 it was those older than 60 and for 2011–2012 it was only the males aged 70+ that did not meet the total DV. With only one exception (40–49 age group in the 2001–2002 NHANES survey) females in all survey years and age groups did not meet the total DV (Figure 1).

Because Se was the lowest value, the ratios were calculated relative to the Se intake (Se = 1). The DV ratio suggests that for every 1 mg of Se, males and females aged 50–59 years should be consuming 85,455 mg of K; 41,818 mg of Na; 23,636 mg of Ca; 22,727 mg of P; 7636 mg of Mg; 327 mg of Zn; 200 mg of Fe and 16 mg of Cu (Table 1). For the DRIs this would mean for every 1 mg of Se, males should be consuming 85,455 mg of K; 23,636 mg of Na; 18,182 mg of Ca; 12,727 mg of P; 7636 mg of Mg; 200 mg of Zn; 145 mg of Fe and 16 mg of Cu (Table 1). However, males aged 50–59 consumed 23,667 mg of K; 30,097 mg of Na; 11,561 mg of Ca; 7506 mg of P; 2577 mg of Mg; 131 mg of Zn; 112 mg of Fe and 12 mg of Cu, for every 1 mg of Se from 2007–2008 (NHANES 2007–2008 participants). For the 2013–2014 NHANES survey year, 22,135 mg of K; 28,996 mg of Na; 11,310 mg of Ca; 7210 mg of P; 2562 mg of Mg; 121 mg of Zn; 92 mg of Fe and 11 mg of Cu, were consumed for every 1 mg of Se. On the other hand, females aged 50–59 years, because the DRI for Ca is higher (1200 mg versus 1000 mg), are recommended to have 85,455 mg of K; 23,636 mg of Na; 21,818 mg of Ca; 12,727 mg of P; 5818 mg of Mg; 145 mg of Zn; 145 mg of Fe and 16 mg of Cu for every 1 mg of Se.

Nonetheless, females, aged 50–59, from NHANES 2007–2008 consumed 28,230 mg of K; 32,478 mg of Na; 13,031 mg of Ca; 9569 mg of P; 3119 mg of Mg; 146 mg of Zn; 111 mg of Fe and 14 mg of Cu for every 1 mg of Se. For every 1 mg of Se, females aged 50–59 years from the NHANES 2013–2014 survey consumed 24,614 mg of K; 29,804 mg of Na; 12,122 mg of Ca; 8527 mg of P; 2863 mg of Mg; 135 mg of Zn; 94 mg of Fe and 11 mg of Cu (Table 1). The ratios of minerals at meal occasions (breakfast, lunch, dinner and snacks) from *What We Eat in America* data tables were also assessed. It is reasonable to expect differences in nutrient ratios at each meal occasion. Table 2 shows the most minerals are consumed at dinner but in general, meal differences are related to K, Na, Ca and P intakes.

Mineral intakes for each of the survey years (2007–2008 and 2013–2014) are also represented as pie charts for males (Figure S1) and females (Figure S2) aged 50–59 years, as well as the DRIs and DVs.

It is possible that many of the NHANES participants, especially females met the DRI for Ca and other minerals through supplementation; however, the focus was on food intakes. Including supplements in ratio calculations adds another dimension to dietary intakes but may be disconnected when correlating with food patterns. It is an interesting area of research to peruse and conceivably urgent as supplement use is reported by over 50% of NHANES respondents, as well as shifting patterns in supplement type, less multi-vitamin-mineral use and more single nutrients (vitamin D) [38]. The influence of supplements on dietary intake ratios would also be potentially fascinating.

### 3.2. Vitamin Ratios

**Figure 2:**
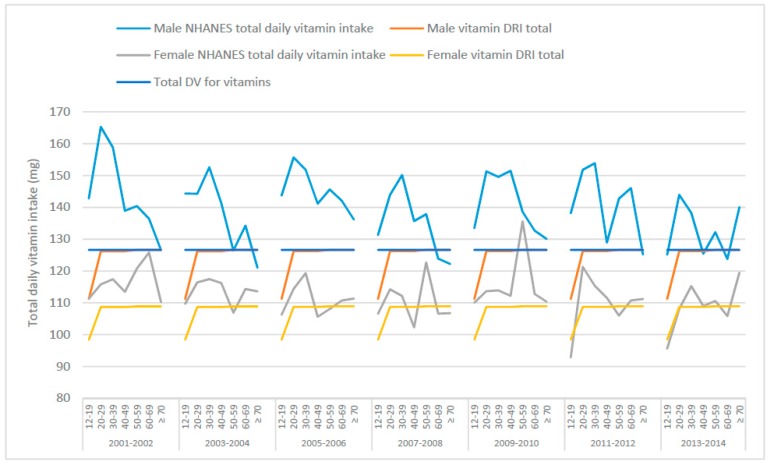
Total mean daily vitamin intakes from food across various age groups, derived from National Health and Nutrition Examination Survey (NHANES) 2001–2014 data, the total Dietary Reference Intakes (DRI) and the total Daily Value (DV) for males and females.

Regarding total vitamin intakes from food (Figure 2 and Table 3), males in general seem to exceed the total mineral intake suggested by the DRIs and DVs. For females, because the DRIs and DVs differ, the DRIs are exceeded in most instances while the intakes are lower than the DVs, especially the 2016 DVs. Assuming the DRIs for vitamins C, B_3_, E, B_6_, B_2_, B_1_, A, folate and vitamins K, D and B_12_ were ingested, males 50–59 years would be consuming approximately 127 mg of these vitamins per day, whereas females 50–59 years would be consuming approximately 109 g of the same vitamins (Table 3). If Americans adhered to the DVs for vitamins, males and females 50–59 years would be receiving approximately 127 mg per day (Table 3). NHANES summary data (from What We Eat In America [37]) from 2007–2008 showed that approximately 138 mg and 123 mg of vitamins were consumed by males and females, respectively, from food in one day. Daily intakes in 2013–2014 were slightly lower compared to 2007–2008 intakes at 132 mg total vitamin intake for males and 111 mg for females (Table 3). On the surface, these data show that total vitamin intakes in the US are just above the DRI and DV totals. Vitamin intakes for each of the survey years (2007–2008 and 2013–2014) are also represented as pie charts for males (Figure S3) and females (Figure S4) aged 50–59 years, as well as the DRIs and DV.

Vitamin B_12_ was set at one (B_12_ = 1), as it was the lowest value. Therefore, for males the DRI suggests that for every mg of B_12_, 37,500 mg of C; 6667 mg of B_3_; 6250 mg of E; 708 mg of B_6_; 542 mg of B_2_; 500 mg of B_1_; 375 mg of A; 167 mg of folate; 50 mg of Vit K and 6 mg of D should be consumed. The DVs have the same values as the male DRIs, relative to B_12_, except for vitamin D, which is 8 mg, not 6 mg, for every mg of B_12_. However, for NHANES 2007–2008 males consumed 14,861 mg of C; 4878 mg of B_3_; 1436 mg of E; 367 mg of B_6_; 416 mg of B_2_; 303 mg of B_1_; 108 mg of A; 96 mg of folate; 21 mg of Vit K and 1 mg of D for every mg of B_12_, while in NHANES 2013–2014 for every mg of B_12_ males consumed 14,946 mg of C; 5394 mg of B_3_; 1864 mg of E; 446 mg of B_6_; 455 mg of B_2_; 332 mg of B_1_; 120 mg of A; 110 mg of folate; 23 mg of Vit K and 1 mg of D (Table 3).

For females, the DRI vitamin ratios, relative to B_12_ differ because the vitamin C recommendations is lower compared to males. Therefore, for each mg of B_12_ females should consume 31,250 mg of C; 5833 mg of B_3_; 6250 mg of E; 625 mg of B_6_; 458 mg of B_2_; 458 mg of B_1_; 292 mg of A; 167 mg of folate; 38 mg of Vit K and 6 mg of D. Females from NHANES 2007–2008 consumed 20,208 mg of C; 4907 mg of B_3_; 1806 mg of E; 412 mg of B_6_; 456 mg of B_2_; 331 mg of B_1_; 142 mg of A; 109 mg of folate; 24 mg of Vit K and 1 mg of D every mg of B_12_ and the NHANES 2013–2014 vitamin ratios showed that for every mg of B_12_ females consumed 19,866 mg of C; 5791 mg of B_3_; 2306 mg of E; 483 mg of B_6_; 507 mg of B_2_; 373 mg of B_1_; 160 mg of A; 122 mg of folate; 38 mg of Vit K and 1 mg of D (Table 3). The ratios of vitamins at meal occasions are shown in Table 4. Again, larger differences were expected between survey years, yet differences were minor suggesting similar dietary patterns/nutrient ratios are present for many years.

As mentioned previously, supplements were not included in this analysis, as micronutrients from food were the focus due to the easier connection to food patterns. Supplement use and their contribution to healthy eating patterns, in addition to how they change micronutrient ratios are areas for future research, especially if healthy micronutrient ratios are established; supplements then could have a direct benefit.

**Figure 3:**
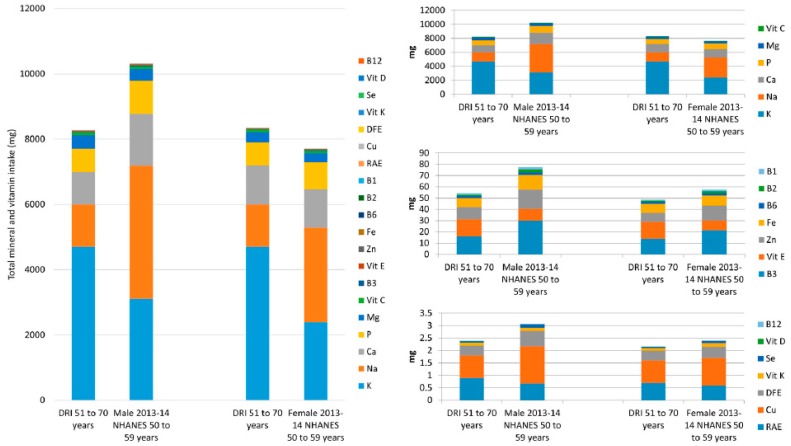
Male and female, aged 50–59 years, mean daily mineral and vitamin intakes from food, from National Health and Nutrition Examination Survey (NHANES) 2013–2014 data versus the DRI. The large figure includes all the minerals and vitamins combined; the smaller figures to the right contain smaller numbers of micronutrients for detail. Abbreviations: Abbreviations: calcium (Ca), phosphorus (P), magnesium (Mg), iron (Fe), zinc (Zn), copper (Cu), selenium (Se), potassium (K), sodium (Na), vitamin A (as retinol activity equivalents (RAE)), thiamin (B_1_), riboflavin (B_2_), niacin (B_3_), pyridoxine (B_6_), cobalamin (B_12_), folate (as dietary folate equivalent (DFE)), vitamin C (Vit C), vitamin D (Vit D), vitamin E (Vit E) and vitamin K (Vit K).

Table 5 contains the data for the minerals and vitamins combined, from food, for males and females, aged 50–59, from the same survey years. The DRIs for minerals (K, Na, Ca, P, Mg, Zn, Fe, Cu and Se) and vitamins (C, B_3_, E, B_6_, B_2_, B_1_, RAE, DFE, Vit K, D and B_12_) combined are 8267 mg and 8346 mg for males and females aged 51–70, respectively. The DV total for the combined vitamins and minerals discussed is 10,127 mg. NHANES data for 50–59-year old’s, from 2013–2014 show that males had a total daily mineral and vitamin intake of 10,310 mg and females had an intake of 7701 mg. This shows that in the most recent NHANES survey, males obtained more than the total DRI mineral and vitamin intake whereas females did not (Table 5 and Figure 3). Figure 3 contains all the mineral and vitamin intakes from food in a stacked column, as well as three smaller columns with nutrients segmented. The nutrients with the six largest intake values (K, Na, Ca, P, Mg and vitamin C) have a DRI (51–70 year olds) total of 8210 mg for males and 8295 mg for females, for these micronutrients, and account for over 99% of the total intake. The NHANES 2013–2014 totals were 10,229 mg and 7641 mg for males and females 50–59 years respectively, showing males intakes were above the DRI value and females below, from food. Next are the nutrients whose DRIs range from 16–1.2 mg/day (B_3_, Vit E, Zn, Fe, B_6_, B_2_ and B_1_). While these account for less than 1% of the total, the total DRI (males and females aged 51–70) for these nutrients combined are 54 mg and 49 mg respectively. However, NHANES 2013–2014 data showed that intakes were 77 mg for males aged 50–59 and 57 mg for females of the same ages, showing both males and females exceed the DRI total for these nutrients from food. The remaining micronutrients (A, Cu, DFE, K, Se, D and B_12_), have a total combined DRI intake of 2.4 mg for males 51–70 years and 2.2 mg for females aged 51–70, whereas NHANES intakes from 2013–2014 were 3.1 mg and 2.4 mg for males and females, aged 50–59 respectively, again showing males and females exceed the DRI total for these nutrients, from food.

These data for the combination of minerals and vitamins show that even though females have an overall insufficient intake for the micronutrients listed above, according to DRI, the insufficiency is overtly apparent for the six nutrients with the largest values (K, Na, Ca, P, Mg and vitamin C). As already mentioned, supplement use by NHANES participants may reduce this number; however, food patterns and not supplement use are the cornerstone of dietary guidelines. Micronutrient patterns/ratios must first be established before the benefits of supplements can be recognized.

## 4. Discussion

If this paper were an analysis of a single nutrient such as Ca, we would conclude that women aged 50–59 years have an insufficient intake, whereas males are meeting the DRI from food. Alternatively, if we were analyzing B_12_ we would have shown that the average intake from food, for males and females aged 50–59, are well above the DRI. However, we would not have shown that the same women are insufficient in Ca but sufficient in B_12_ and males have a sufficient intake of both from food. We show that by examining micronutrient patterns a different picture of diet quality emerges.

For the minerals, the most obvious difference (Figures S1 and S2 and Table 1) between recommendations and intakes is the K to Na ratio, even though the overall patterns from NHANES look very different from the DRI (Figures S1 and S2). For 50–59 year olds, the DRI K to Na ratio is 4/1, whereas the DV has a ratio of 2/1. However, in the 2007–2008 and 2013–2014 survey, males and females (50–59 years) had a ratio of 1/1. While these two minerals are a good example, the results imply that minerals are being consumed in the wrong ratio in the US diet (a proxy for the Western diet), if we use the DRIs and DVs as the standard. Moreover, utilizing ratios may add more perspective to DRIs. For example, the DRI for Ca could be 1000 mg because the intake of Na was high (or low Mg, or other combinations) in the populations that were studied, or it could be 1000 mg regardless of the intake of other minerals. An incorrect ratio of mineral intakes may explain why CVD risk is associated with Ca supplement use [39,40]; here the much higher intake of Ca may be affecting the ratios of the other minerals and possibly their physiological actions. The recent associations between a high P intake and all-cause mortality [41] and cardiovascular disease [42] may be due to a disruption in the ratio of P to other nutrients. Furthermore, the ratio of Ca to Mg is well known and recent epidemiological evidence suggests it may be important to determine the ideal ratio to reduce mortality [43] and reduce the risk for postmenopausal breast cancer [44]. This would then suggest mineral research might benefit by shifting to investigating ratios of minerals on health outcomes as opposed to individual mineral levels. However, it is possible that optimal/ideal mineral ratios will turn out to be sex, age and possibly more importantly for clinical nutrition, disease specific. The optimal ratio of minerals required to promote health, athletic activity, healthy aging, or prevent/treat conditions is unknown. For future mineral studies, nutritional scientists may have to consider the ratio of minerals in the diet, or evaluate blood/tissue by chemical analysis, before arriving at conclusions (especially related to clinical endpoints).

For the vitamins, the differences in ratios between DRIs, DVs and NHANES intakes is less noticeable compared to the minerals but does exist (Figures S3 and S4 and Table 3) The fat-soluble vitamins account for only 13% (16 mg) of the overall total daily intake for males and 14% (16 mg) for females. Even so, we have previously shown that the intake of fat soluble vitamins are chronically insufficient in the US diet [36]. Similar to the minerals, vitamin ratios may help explain why the DRIs are at their current levels, for example; the DRI for vitamin E could be 15 mg because the intake of vitamin C is 90 mg. To highlight differences between the DRIs/DVs and NHANES intakes some simpler examples can be used and these examples are for illustrative purposes only. A potentially interesting value may be the ratio of water soluble to fat soluble vitamins. Using the DRIs this ratio should be 41/1 for males and 33/1 for females, the DV ratio is 41/1. The 2007–2008 NHANES data (from What We Eat In America summary tables) showed that the male and female ratios were 55/1 and 45/1, respectively, for the 2007–2008 survey, and, 48/1 and 41/1 for male and female ratios, respectively. This shows that more water-soluble vitamins are being consumed relative to what is recommended. For another example, the ratio of the major antioxidant nutrients (C/E/Se) may show differences. Relative to Se, the DRIs for 50–70 year olds provide ratios of 1636/273/1 for males and 1364/273/1 for females. However, NHANES 2013–2014 derived data show that the relative C/E/Se ratios were 594/74/1 and 763/89/1 for males and females 50–59 years, respectively. The ratios of minerals and vitamins at meal occasions indicated minor differences between survey years, approximately 6 years apart. Although some observable differences were expected between survey years, minor changes suggest similar dietary patterns/nutrient ratios are present in the US diet for many years. The importance and use of vitamin ratios is yet to be discovered; however, ratios of smaller numbers of vitamins may provide some information regarding how vitamin ratios affect metabolism and health outcomes.

Although this was not a statistical analysis, the observed lack of major differences between micronutrient intakes at each eating occasion was not expected. However, the effects, if any, of these minor differences are unknown. One possible explanation is that because the 2015 DG and previous dietary guidelines, impact food policy it could be that more and more foods/meals/supplements are formulated to meet the DRIs and DVs. This could mean the meal-to-meal variation in nutrient intakes is lost. It may be interesting to explore the nutrient ratios at each meal for those people consuming whole foods only, versus a diet higher in processed foods to see if higher meal-to-meal nutrient variation is present and beneficial. The DRIs and DVs however, may apply to the formulation of sole source nutritional products (as used in tube feeding and other special circumstances), nevertheless, it must be remembered that a product formulated to meet the DRIs is essentially designed to avoid deficiency. The DRIs are not disease or condition specific and disease conditions may change the requirements for micronutrients [36]; this presents a large research gap for nutrition.

Nutrient intake ratios were discussed based on NHANES intakes and DRIs, however, there is no indication of what a harmful micronutrient ratio may be. Tolerable upper intake levels (ULs) exist for P, Na, Ca, Vit C, Vit E, B_6_, Fe, Zn, B_3_, Cu, RAE, DFE, Se and Vit D and their ratios were calculated (Table 6). Mg was not included as the UL is only for pharmacological agents and not from food sources. It is rare for an individual to have all these nutrient intakes at or above the ULs; however, it may be possible to use the UL ratios as a cut off. As an example, we have provided a hypothetical diet in Table 6 that contains micronutrients at the DRI but with 2000 mg of vitamin C. The ratio values changed showing nutrient ratios change, as did the value relative to vitamin D (from 6000 to 133,333). While choline is not considered a true vitamin, it is an essential nutrient with numerous important functions [45] and the 2015 DG indicate it is a nutrient of concern, however for simplicity it was not included in this paper. It is a nutrient that can be considered in future ratio based analyses.

One final topic must be covered regarding micronutrient ratios. Within micronutrient ratios there is the concept of proportionality. Simply put, if the proportion of Na and K is 1 g of Na for every 4 g of K, it may not matter if one consumes 10 g of sodium once the amount of K consumed is 40 g. Physiologically, we expect relationships of micronutrients to be a mixture of linear, exponential and potentially saturable. However, for purposes of illustration, having the proportions of the micronutrients within the DRIs be ideal, the micronutrient intakes from NHANES would change for males and females aged 50–59 years based on their total intakes from 2013–2014 NHANES summary data. Table 7 shows how vitamin and mineral intakes would change, if the total (NHANES reported) intakes were in proportion to the DRI ratios. The greatest differences are in the six micronutrients required in the greatest amounts (K, Na, Ca, P, Mg and Vit C). Because males had a greater total micronutrient intake from food than the DRI (10,310 mg v. 8267 mg), all minerals and vitamins are greater than but still in proportion to the DRIs, whereas for females the total intake were below the DRIs (7701 mg v. 8346 mg) resulting in all the micronutrients being in proportion to but not meeting, the DRI. Supplements, single or multi-nutrient, could fill the gaps but it would be difficult to have a supplement with the correct concentrations to adjust for gaps in the daily micronutrient ratio, if established, especially if excesses are present. Nevertheless, supplements play a role to in the modern diet and efforts should be made to integrate them into dietary patterns.

An important point to consider when advocating dietary patterns over focusing on DRIs, or even optimal levels of single nutrients, is that dietary patterns conceivably account for occasional, or even regular, poor dietary choices. A good dietary pattern may not meet the DRI for every nutrient. If this is the case then it is possible that a good dietary pattern includes a certain percentage of poor food choices and days not meeting DRIs. Conversely, it may be that, not adhering strictly to the DRIs each and every day may be related to chronic disease risk.

While the total micronutrient intakes are sufficient in the US population, it may be their ratios that are out of balance and ultimately contribute to syndromes such as osteosarcopenic obesity [36]. A recent analysis of the Korean National Health and Nutritional Examination Survey (2008–2010) showed that women with a better diet quality were less likely to have osteosarcopenic obesity [46]. Although this was not found in males there may be subtle differences in dietary patterns between males and females which may be detected using nutrient ratios.

The classic method of nutrient research is to change the concentration of one specific nutrient and measure outcomes, or use large databases to obtain retrospective observational data, with various statistical adjustments, to associate a food or nutrient with an outcome. Both of these approaches may not take into consideration the dynamic multifunctional nature of the nutrient in question and its relationship to other nutrients. The focus on dietary patterns in the new 2015 DG makes sense because that is how food is consumed; however, cultural differences may limit the use of standard dietary patterns globally. Williams et al. support dietary pattern type research but also call for more studies with whole diet interventions suitable for real world studies, compliance being the primary obstacle for most studies, but also individual differences in response to diet [47]. Leite proposes applying compositional methodology to nutritional epidemiology [48], which may be able to separate individual dietary components and adjust for energy intake and this type of analysis may be appropriate for all nutritional studies. A recent article, based on a dairy research expert workshop in Denmark, suggests evidence from single nutrients be combined with whole food studies to form future dietary recommendations [49].

A new method to look at diet and health outcomes in a holistic fashion are Nutrient-Wide Association Studies [50] with methodology borrowed from Environmental-Wide Association Studies [51]; both analyses are rooted in Genome-wide Association Studies with environmental factors, or nutrients, replacing genetic loci. Tzoulaki et al. searched for associations between 82 dietary components (and 3 urinary minerals) and blood pressure, using data from the International Population Study on Macronutrients and Blood Pressure (INTERMAP) and NHANES [50]. Results showed that the intakes of several B vitamins were negatively correlated with blood pressure, as well as reinforcing the importance of the Na/K ratio. While there is room for interpretation of the results due to differences between INTERMAP and NHANES data, these types of studies are a positive step forward for nutritional science and strengthen the need for new, inclusive, systems based, methodology in nutrition (basic and clinical). Results also suggest that dietary ratios of B vitamins (folate, riboflavin and thiamin) may be important in the development of coronary heart disease. However, results from these types of studies are primarily observational and provide hypothesis for later follow up in controlled clinical trials; the issue is how to design randomized clinical trials, with appropriate controls, to investigate for cause-effect. While the concept of nutrient ratios is applicable to observational and interventional studies, the major limitation is the lack of dietary intake data and nutrient biomarkers to coincide with other endpoints. Because seemingly minor differences in intake patterns over meal occasions and overall were seen in this data, there is the possibility that minor differences may represent a nutritional butterfly effect [52] to the overall diet in relation to the outcome measured.

It may also be time to include disease endpoints in dietary intervention trials [47] and the recent guiding principles from the National Academies of Sciences, Engineering and Medicine are a major step forward in having future DRIs specific to chronic diseases [53] (based on a working group report [54]). One benefit of using disease endpoints is that nutrition will become more important in the healthcare setting and the potential for dietary assessment becoming routine for clinicians, however, to track dietary related disease progression is a large and lengthy undertaking. For all encompassing dietary guidelines targeted at maintaining good nutritional status, other endpoints are also required—e.g., optimal bone mass, muscle mass, minimizing fat mass accrual, cognition, enhancing gut microflora. Other basic science endpoints are also required to find nutrient intakes that optimize human metabolism. Micronutrient intakes from diet are the focus of this paper, however the same principles could be applied to mineral and vitamin biomarker ratios.

The concept of nutrient ratios may bridge the gap between single nutrient studies and dietary patterns. The focus of this analysis was on those aged 14 years or older, because these ages would be expected to make more individual food choices, however, micronutrient ratios could apply to younger ages as well as pediatric/infant nutrition. Micronutrient ratios are not meant to replace other forms of epidemiological research or appropriate single nutrient studies; rather they can coexist and may even complement each other. We anticipate this introduction to the concept of micronutrient ratios, whether it be for sets of related micronutrients or all micronutrients, to be the catalyst for scientific discussion and possibly stimulate new ideas in nutritional science and promote a change in the way nutrients are regarded and studied.

## 5. Conclusions

This paper has shown that physiologically micronutrients are rarely consumed individually and metabolism, in all its complexity, is a cornucopia of ratios. The commitment to investigating micronutrients as single entities may be the reason nutritional studies outcomes are so varied. A fundamental rethinking of nutritional science is necessary to address how dietary patterns (a complex ratio of nutrients) affect health and clinical outcomes and how they can translate to individual recommendations. A dietary ratio of micronutrients (for healthy people) is already inherent in all diet patterns as well as the DRIs and these could be the standard for future studies that measure the effects of micronutrient ratios on health outcomes. However, much more work is required; including new mathematical methods and study designs before nutrient ratios can be fully utilized for nutritional studies.

## Figures and Tables

**Table 1:** The Dietary Reference Intakes (DRI), Daily Value (DV) and dietary mineral intakes from food (National Health and Nutrition Examination Survey (NHANES) 2007–2008 and 2013–2014 data), their ratios and their concentration relative to calcium, in males and females 51–59 years.

Data Set	K	Na	Ca	P	Mg	Zn	Fe	Cu	Se	Total
DV value (mg)	4700	2300	1300	1250	420	18	11	0.9	0.055	9999.9550
DV ratio	4.70 × 10^−1^	2.30 × 10^−1^	1.30 × 10^−1^	1.25 × 10^−1^	4.20 × 10^−2^	1.80 × 10^−3^	1.10 × 10^−3^	9.00 × 10^−5^	5.50 × 10^−6^	1
DV ratio with Se set at 1	85,455	41,818	23,636	22,727	7636	327	200	16	1	
Male										
DRI 51–70 years (mg)	4700	1300	1000	700	420	11	8	0.9	0.055	8139.9550
DRI 51–70 years ratio	5.77 × 10^−1^	1.60 × 10^−1^	1.23 × 10^−1^	8.60 × 10^−2^	5.16 × 10^−2^	1.35 × 10^−3^	9.83 × 10^−4^	1.11 × 10^−4^	6.76 × 10^−6^	1
DRI 51–70 years ratio with Se set at 1	85,455	23,636	18,182	12,727	7636	200	145	16	1	
Male NHANES 2007–2008 mean intake, 50–59 years (mg)	3169	4030	1005	1548	345	15	17.6	1.6	0.1339	10,131.3339
Male NHANES 2007–2008 mean intake, 50–59 years ratio	3.13 × 10^−1^	3.98 × 10^−1^	9.92 × 10^−2^	1.53 × 10^−1^	3.41 × 10^−2^	1.48 × 10^−3^	1.74 × 10^−3^	1.58 × 10^−4^	1.32 × 10^−5^	1
Male NHANES 2007–2008 mean intake, 50–59 years ratio with Se set at 1	23,667	30,097	7506	11,561	2577	112	131	12	1	
Male NHANES 2013–2014 mean intake, 50–59 years (mg)	3110	4074	1013	1589	360	12.9	17	1.5	0.1405	10,177.5405
Male NHANES 2013–2014 mean intake, 50–59 years ratio	3.06 × 10^−1^	4.00 × 10^−1^	9.95 × 10^−2^	1.56 × 10^−1^	3.54 × 10^−2^	1.27 × 10^−3^	1.67 × 10^−3^	1.47 × 10^−4^	1.38 × 10^−5^	1
Male NHANES 2013–2014 mean intake, 50–59 years ratio with Se set at 1	22,135	28,996	7210	11,310	2562	92	121	11	1	
Female										
DRI 51–70 years (mg)	4700	1300	1200	700	320	8	8	0.9	0.055	8236.9550
DRI 51–70 years ratio	5.70 × 10^−1^	1.58 × 10^−1^	1.46 × 10^−1^	8.50 × 10^−2^	3.88 × 10^−2^	9.71 × 10^−4^	9.71 × 10^−4^	1.09 × 10^−4^	6.68 × 10^−6^	1
DRI 51–70 years ratio with Se set at 1	85,455	23,636	21,818	12,727	5818	145	145	16	1	
Female NHANES 2007–2008 mean intake, 50–59 years (mg)	2552	2936	865	1178	282	10	13.2	1.3	0.0904	7837.5904
Female NHANES 2007–2008 mean intake, 50–59 years ratio	3.26 × 10^−1^	3.75 × 10^−1^	1.10 × 10^−1^	1.50 × 10^−1^	3.60 × 10^−2^	1.28 × 10^−3^	1.68 × 10^−3^	1.66 × 10^−4^	1.15 × 10^−5^	1
Female NHANES 2007–2008 mean intake, 50–59 years ratio with Se set at 1	28,230	32,478	9569	13,031	3119	111	146	14	1	
Female NHANES 2013–2014 mean intake, 50–59 years (mg)	2390	2894	828	1177	278	9.1	13.1	1.1	0.0971	7590.3970
Female NHANES 2013–2014 mean intake, 50–59 years ratio	3.15 × 10^−1^	3.81 × 10^−1^	1.09 × 10^−1^	1.55 × 10^−1^	3.66 × 10^−2^	1.20 × 10^−3^	1.73 × 10^−3^	1.45 × 10^−4^	1.28 × 10^−5^	1
Female NHANES 2013–2014 mean intake, 50–59 years ratio with Se set at 1	24,614	29,804	8527	12,122	2863	94	135	11	1	

Values are rounded. Abbreviations: calcium (Ca), phosphorus (P), magnesium (Mg), iron (Fe), zinc (Zn), copper (Cu), selenium (Se), potassium (K) and sodium (Na).

**Table 2:** The mineral intakes (National Health and Nutrition Examination Survey (NHANES) 2007–2008 and 2013–2014 data), their ratios and their ratios relative to selenium (Se) at meal occasions, for males and females 51–59 years.

Data Set	K	Na	Ca	P	Mg	Zn	Fe	Cu	Se
Breakfast									
Male NHANES 2007–2008 mean intake (mg)	590.90	611.10	212.73	301.91	64.80	2.06	4.93	0.23	0.02
Male NHANES 2007–2008 mean intake, 50–59 years ratio	3.31 × 10^−1^	3.42 × 10^−1^	1.19 × 10^−1^	1.69 × 10^−1^	3.62 × 10^−2^	1.15 × 10^−3^	2.76 × 10^−3^	1.26 × 10^−4^	1.25 × 10^−5^
Male NHANES 2007–2008 mean intake, 50–59 years ratio with Se set at 1	26,286	27,184	9463	13,430	2,883	92	219	10	1
Female NHANES 2007–2008 mean intake (mg)	454.10	405.16	182.16	235.40	55.60	1.73	3.54	0.18	0.02
Female NHANES 2007–2008 mean intake, 50–59 years ratio	3.39 × 10^−1^	3.03 × 10^−1^	1.36 × 10^−1^	1.76 × 10^−1^	4.16 × 10^−2^	1.29 × 10^−3^	2.64 × 10^−3^	1.32 × 10^−4^	1.16 × 10^−5^
Female NHANES 2007–2008 mean intake, 50–59 years ratio with Se set at 1	29,229	26,079	11,725	15,152	3579	111	228	11	1
Male NHANES 2013–2014 mean intake (mg)	665.49	644.80	231.15	309.60	65.55	3.15	5.28	0.30	0.02
Male NHANES 2013–2014 mean intake, 50–59 years ratio	3.46 × 10^−1^	3.35 × 10^−1^	1.20 × 10^−1^	1.61 × 10^−1^	3.40 × 10^−2^	1.64 × 10^−3^	2.74 × 10^−3^	1.58 × 10^−4^	1.25 × 10^−5^
Male NHANES 2013–2014 mean intake, 50–59 years ratio with Se set at 1	27,611	26,753	9590	12,845	2720	131	219	13	1
Female NHANES 2013–2014 mean intake (mg)	561.44	411.04	216.25	247.38	62.04	2.10	3.70	0.23	0.02
Female NHANES 2013–2014 mean intake, 50–59 years ratio	3.73 × 10^−1^	2.73 × 10^−1^	1.44 × 10^−1^	1.64 × 10^−1^	4.12 × 10^−2^	1.40 × 10^−3^	2.46 × 10^−3^	1.56 × 10^−4^	1.02 × 10^−5^
Female NHANES 2013–2014 mean intake, 50–59 years ratio with Se set at 1	36,533	26,746	14,071	16,097	4037	137	240	15	1
Lunch									
Male NHANES 2007–2008 mean intake (mg)	715.30	1222.20	243.12	397.25	79.20	4.13	3.91	0.41	0.04
Male NHANES 2007–2008 mean intake, 50–59 years ratio	2.68 × 10^−1^	4.59 × 10^−1^	9.12 × 10^−2^	1.49 × 10^−1^	2.97 × 10^−2^	1.55 × 10^−3^	1.47 × 10^−3^	1.52 × 10^−4^	1.58 × 10^−5^
Male NHANES 2007–2008 mean intake, 50–59 years ratio with Se set at 1	16,970	28,996	5768	9425	1879	98	93	10	1
Female NHANES 2007–2008 mean intake	525.80	839.26	173.88	282.48	55.60	2.09	3.01	0.24	0.03
Female NHANES 2007–2008 mean intake, 50–59 years ratio	2.79 × 10^−1^	4.46 × 10^−1^	9.24 × 10^−2^	1.50 × 10^−1^	2.95 × 10^−2^	1.11 × 10^−3^	1.60 × 10^−3^	1.29 × 10^−4^	1.44 × 10^−5^
Female NHANES 2007–2008 mean intake, 50–59 years ratio with Se set at 1	19,339	30,869	6395	10,390	2045	77	111	9	1
Male NHANES 2013–2014 mean intake	665.49	1007.50	211.05	340.56	65.55	3.45	3.34	0.32	0.03
Male NHANES 2013–2014 mean intake, 50–59 years ratio	2.90 × 10^−1^	4.39 × 10^−1^	9.19 × 10^−2^	1.48 × 10^−1^	2.85 × 10^−2^	1.50 × 10^−3^	1.46 × 10^−3^	1.39 × 10^−4^	1.51 × 10^−5^
Male NHANES 2013–2014 mean intake, 50–59 years ratio with Se set at 1	19,116	28,940	6062	9782	1883	99	96	9	1
Female NHANES 2013–2014 mean intake	586.96	910.16	198.95	294.50	59.22	2.30	2.77	0.27	0.03
Female NHANES 2013–2014 mean intake, 50–59 years ratio	2.86 × 10^−1^	4.43 × 10^−1^	9.68 × 10^−2^	1.43 × 10^−1^	2.88 × 10^−2^	1.12 × 10^−3^	1.35 × 10^−3^	1.33 × 10^−4^	1.31 × 10^−5^
Female NHANES 2013–2014 mean intake, 50–59 years ratio with Se set at 1	21,643	33,560	7336	10,859	2184	85	102	10	1
Dinner									
Male NHANES 2007–2008 mean intake	1181.80	1711.08	314.03	619.71	122.40	5.03	5.95	0.53	0.06
Male NHANES 2007–2008 mean intake, 50–59 years ratio	2.98 × 10^−1^	4.32 × 10^−1^	7.93 × 10^−2^	1.56 × 10^−1^	3.09 × 10^−2^	1.27 × 10^−3^	1.50 × 10^−3^	1.33 × 10^−4^	1.63 × 10^−5^
Male NHANES 2007–2008 mean intake, 50–59 years ratio with Se set at 1	18,286	26,475	4859	9589	1894	78	92	8	1
Female NHANES 2007–2008 mean intake	860.40	1244.42	248.40	435.49	88.96	3.64	4.45	0.37	0.04
Female NHANES 2007–2008 mean intake, 50–59 years ratio	2.98 × 10^−1^	4.31 × 10^−1^	8.61 × 10^−2^	1.51 × 10^−1^	3.08 × 10^−2^	1.26 × 10^−3^	1.54 × 10^−3^	1.30 × 10^−4^	1.48 × 10^−5^
Female NHANES 2007–2008 mean intake, 50–59 years ratio with Se set at 1	20,139	29,127	5814	10,193	2082	85	104	9	1
Male NHANES 2013–2014 mean intake	1204.22	1894.10	321.60	603.72	117.30	6.00	6.16	0.54	0.06
Male NHANES 2013–2014 mean intake, 50–59 years ratio	2.90 × 10^−1^	4.56 × 10^−1^	7.74 × 10^−2^	1.45 × 10^−1^	2.82 × 10^−2^	1.44 × 10^−3^	1.48 × 10^−3^	1.31 × 10^−4^	1.45 × 10^−5^
Male NHANES 2013–2014 mean intake, 50–59 years ratio with Se set at 1	19,985	31,435	5337	10,019	1947	100	102	9	1
Female NHANES 2013–2014 mean intake	867.68	1203.76	233.55	412.30	84.60	3.90	4.36	0.39	0.04
Female NHANES 2013–2014 mean intake, 50–59 years ratio	3.09 × 10^−1^	4.28 × 10^−1^	8.31 × 10^−2^	1.47 × 10^−1^	3.01 × 10^−2^	1.39 × 10^−3^	1.55 × 10^−3^	1.39 × 10^−4^	1.38 × 10^−5^
Female NHANES 2013–2014 mean intake, 50–59 years ratio with Se set at 1	22,321	30,967	6008	10,607	2176	100	112	10	1
All snacks									
Male NHANES 2007–2008 mean intake	653.10	529.62	243.12	270.13	93.60	1.68	2.21	0.36	0.01
Male NHANES 2007–2008 mean intake, 50–59 years ratio	3.64 × 10^−1^	2.95 × 10^−1^	1.36 × 10^−1^	1.51 × 10^−1^	5.22 × 10^−2^	9.35 × 10^−4^	1.23 × 10^−3^	2.01 × 10^−4^	6.27 × 10^−6^
Male NHANES 2007–2008 mean intake, 50–59 years ratio with Se set at 1	58,105	47,119	21,630	24,033	8327	149	197	32	1
Female NHANES 2007–2008 mean intake	573.60	405.16	223.56	235.40	80.62	1.64	2.10	0.31	0.01
Female NHANES 2007–2008 mean intake, 50–59 years ratio	3.78 × 10^−1^	2.66 × 10^−1^	1.47 × 10^−1^	1.55 × 10^−1^	5.30 × 10^−2^	1.08 × 10^−3^	1.38 × 10^−3^	2.02 × 10^−4^	7.65 × 10^−6^
Female NHANES 2007–2008 mean intake, 50–59 years ratio with Se set at 1	49,228	34,772	19,186	20,203	6919	141	180	26	1
Male NHANES 2013–2014 mean intake	665.49	483.60	231.15	294.12	96.60	2.40	2.82	0.43	0.01
Male NHANES 2013–2014 mean intake, 50–59 years ratio	3.75 × 10^−1^	2.72 × 10^−1^	1.30 × 10^−1^	1.66 × 10^−1^	5.44 × 10^−2^	1.35 × 10^−3^	1.59 × 10^−3^	2.43 × 10^−4^	8.29 × 10^−6^
Male NHANES 2013–2014 mean intake, 50–59 years ratio with Se set at 1	45,182	32,833	15,694	19,969	6558	163	191	29	1
Female NHANES 2013–2014 mean intake	535.92	381.68	224.90	223.82	78.96	1.60	2.38	0.40	0.01
Female NHANES 2013–2014 mean intake, 50–59 years ratio	3.70 × 10^−1^	2.63 × 10^−1^	1.55 × 10^−1^	1.54 × 10^−1^	5.45 × 10^−2^	1.10 × 10^−3^	1.64 × 10^−3^	2.78 × 10^−4^	6.24 × 10^−6^
Female NHANES 2013–2014 mean intake, 50–59 years ratio with Se set at 1	59,283	42,221	24,878	24,759	8735	177	263	45	1

Values are rounded. Abbreviations: calcium (Ca), phosphorus (P), magnesium (Mg), iron (Fe), zinc (Zn), copper (Cu), selenium (Se), potassium (K) and sodium (Na).

**Table 3:** The Dietary Reference Intakes (DRI), Daily Values (DV) and dietary vitamin intakes from food (derived from National Health and Nutrition Examination Survey (NHANES) 2007–2008 and 2013–2014 data), their ratios and their ratio relative to vitamin B_12_, in males and females 50–59 years.

Data Set	C	B_3_	E	B_6_	B_2_	B_1_	RAE	DFE	Vit K	D	B_12_	Total	Total Water Soluble	Total Fat Soluble
2016 DV value (mg)	90	16	15	1.7	1.3	1.2	0.9	0.4	0.12	0.02	0.0024	126.64	110.60	16.04
DV ratio	7.11 × 10^−1^	1.26 × 10^−1^	1.18 × 10^−1^	1.34 × 10^−2^	1.03 × 10^−2^	9.48 × 10^−3^	7.11 × 10^−3^	3.16 × 10^−3^	9.48 × 10^−4^	1.58 × 10^−5^	1.90 × 10^−5^			
DV ratio with B_12_ set at 1	37,500	6667	6250	708	542	500	375	167	50	8	1			
Males 51–70 years														
DRI (mg)	90	16	15	1.7	1.3	1.2	0.9	0.4	0.12	0.015	0.0024	126.64	110.6024	16.04
DRI ratio	7.11 × 10^−1^	1.26 × 10^−1^	1.18 × 10^−1^	1.34 × 10^−2^	1.03 × 10^−2^	9.48 × 10^−3^	7.11 × 10^−3^	3.16 × 10^−3^	9.48 × 10^−4^	1.18 × 10^−4^	1.90 × 10^−5^			
DRI ratio with B_12_ set at 1	37,500	6667	6250	708	542	500	375	167	50	6	1			
Male NHANES 2007–2008 mean intake (mg)	91.1	29.9	8.8	2.25	2.55	1.86	0.66	0.586	0.1272	0.0053	0.00613	137.84	128.25	9.59
Male NHANES 2007–2008 mean intake ratio	6.61 × 10^−1^	2.17 × 10^−1^	6.38 × 10^−2^	1.63 × 10^−2^	1.85 × 10^−2^	1.35 × 10^−2^	4.79 × 10^−3^	4.25 × 10^−3^	9.23 × 10^−4^	3.84 × 10^−5^	4.45 × 10^−5^			
Male NHANES 2007–2008 mean intake ratio with B_12_ set at 1	14,861	4878	1436	367	416	303	108	96	21	1	1			
Male NHANES 2013–2014 mean intake (mg)	83.4	30.1	10.4	2.49	2.54	1.85	0.672	0.615	0.1305	0.0052	0.00558	132.21	121.00	11.21
Male NHANES 2013–2014 mean intake ratio	6.31 × 10^−1^	2.28 × 10^−1^	7.87 × 10^−2^	1.88 × 10^−2^	1.92 × 10^−2^	1.40 × 10^−2^	5.08 × 10^−3^	4.65 × 10^−3^	9.87 × 10^−4^	3.93 × 10^−5^	4.22 × 10^−5^			
Male NHANES 2013–2014 mean intake ratio with B_12_ set at 1	14,946	5394	1864	446	455	332	120	110	23	1	1			
Females 51–70 years														
DRI (mg)	75	14	15	1.5	1.1	1.1	0.7	0.4	0.09	0.015	0.0024	533.91	518.10	15.81
DRI ratio	6.89 × 10^−1^	1.29 × 10^−1^	1.38 × 10^−1^	1.38 × 10^−2^	1.01 × 10^−2^	1.01 × 10^−2^	6.43 × 10^−3^	3.67 × 10^−3^	8.26 × 10^−4^	1.38 × 10^−4^	2.20 × 10^−5^			
DRI ratio with B_12_ set at 1	31,250	5833	6250	625	458	458	292	167	38	6	1			
Female NHANES 2007–2008 mean intake (mg)	87.3	21.2	7.8	1.78	1.97	1.43	0.614	0.47	0.1058	0.0044	0.00432	122.68	114.15	8.52
Female NHANES 2007–200 mean intake ratio	7.12 × 10^−1^	1.73 × 10^−1^	6.36 × 10^−2^	1.45 × 10^−2^	1.61 × 10^−2^	1.17 × 10^−2^	5.01 × 10^−3^	3.83 × 10^−3^	8.62 × 10^−4^	3.59 × 10^−5^	3.52 × 10^−5^			
Female NHANES 2007–2008 mean intake ratio with B_12_ set at 1	20,208	4907	1806	412	456	331	142	109	24	1	1			
Female NHANES 2013–2014 mean intake (g)	74.1	21.6	8.6	1.8	1.89	1.39	0.597	0.456	0.1416	0.0041	0.00373	110.58	101.24	9.34
Female NHANES 2013–2014 mean intake ratio	6.70 × 10^−1^	1.95 × 10^−1^	7.78 × 10^−2^	1.63 × 10^−2^	1.70 × 10^−2^	1.26 × 10^−2^	5.40 × 10^−3^	4.12 × 10^−3^	1.28 × 10^−3^	3.71 × 10^−5^	3.37 × 10^−5^			
Female NHANES 2013–2014 mean intake ratio with B_12_ set at 1	19,866	5791	2306	483	507	373	160	122	38	1	1			

Values are rounded. Abbreviations: vitamin A (as retinol activity equivalents (RAE)), thiamin (B_1_), riboflavin (B_2_), niacin (B_3_), pyridoxine (B_6_), cobalamin (B_12_), folate (as dietary folate equivalent (DFE)), vitamin C (C), vitamin D (D), vitamin E (E) and vitamin K (Vit K).

**Table 4:** The vitamin intakes from food (National Health and Nutrition Examination (NHANES) 2007–2008 and 2013–2014 data), their ratios and their ratios relative to B_12_ at meal occasions, for males and females 51–59 years.

Data Set	C	B_3_	E	B_6_	B_2_	B_1_	RAE	DFE	Vit K	D	B_12_
Breakfast											
Male NHANES 2007–2008 mean intake (mg)	16.398	5.083	1.496	0.4725	0.612	0.5208	0.1914	0.15822	0.010176	0.001537	0.0014712
Male NHANES 2007–2008 mean intake, 50–59 years ratio	6.57 × 10^−1^	2.04 × 10^−1^	6.00 × 10^−2^	1.90 × 10^−2^	2.45 × 10^−2^	2.10 × 10^−2^	7.70 × 10^−3^	6.34 × 10^−3^	4.08 × 10^−4^	6.16 × 10^−5^	5.90 × 10^−5^
Male NHANES 2007–2008 mean intake, 50–59 years ratio with B12 set at 1	11,146	3455	1017	321	416	354	130	108	7	1	1
Female NHANES 2007–2008 mean intake (mg)	16.587	3.816	1.326	0.4094	0.4728	0.3289	0.17806	0.1269	0.00529	0.00132	0.0012096
Female NHANES 2007–2008 mean intake, 50–59 years ratio	7.13 × 10^−1^	1.64 × 10^−1^	5.70 × 10^−2^	1.76 × 10^−2^	2.03 × 10^−2^	1.41 × 10^−2^	7.66 × 10^−3^	5.46 × 10^−3^	2.27 × 10^−4^	5.68 × 10^−5^	5.20 × 10^−5^
Female NHANES 2007–2008 mean intake, 50–59 years ratio with B12 set at 1	13,713	3155	1096	338	391	272	147	105	4	1	1
Male NHANES 2013–2014 mean intake (mg)	19.182	5.719	2.08	0.5976	0.6096	0.518	0.19488	0.1968	0.01044	0.002028	0.001674
Male NHANES 2013–2014 mean intake, 50–59 years ratio	6.59 × 10^−1^	1.96 × 10^−1^	7.14 × 10^−2^	2.05 × 10^−2^	2.09 × 10^−2^	1.77 × 10^−2^	6.69 × 10^−3^	6.76 × 10^−3^	3.59 × 10^−4^	6.97 × 10^−5^	5.75 × 10^−5^
Male NHANES 2013–2014 mean intake, 50–59 years ratio with B12 set at 1	11,459	3416	1243	357	364	309	116	118	6	1	1
Female NHANES 2013–2014 mean intake (mg)	16.302	4.104	1.634	0.414	0.4536	0.3892	0.19104	0.12312	0.015576	0.001599	0.0010817
Female NHANES 2013–2014 mean intake, 50–59 years ratio	6.90 × 10^−1^	1.74 × 10^−1^	6.92 × 10^−2^	1.75 × 10^−2^	1.92 × 10^−2^	1.65 × 10^−2^	8.09 × 10^−3^	5.21 × 10^−3^	6.59 × 10^−4^	6.77 × 10^−5^	4.58 × 10^−5^
Female NHANES 2013–2014 mean intake, 50–59 years ratio with B12 set at 1	15,071	3794	1511	383	419	360	177	114	14	1	1
Lunch											
Male NHANES 2007–2008 mean intake (mg)	21.864	7.774	2.288	0.54	0.612	0.3534	0.1254	0.12892	0.036888	0.000954	0.0017164
Male NHANES 2007–2008 mean intake, 50–59 years ratio	6.48 × 10^−1^	2.31 × 10^−1^	6.78 × 10^−2^	1.60 × 10^−2^	1.81 × 10^−2^	1.05 × 10^−2^	3.72 × 10^−3^	3.82 × 10^−3^	1.09 × 10^−4^	2.83 × 10^−5^	5.09 × 10^−5^
Male NHANES 2007–2008 mean intake, 50–59 years ratio with B12 set at 1	12,738	4529	1333	315	357	206	73	75	21	1	1
Female NHANES 2007–2008 mean intake (mg)	17.46	5.088	1.638	0.3738	0.4728	0.3146	0.11666	0.1175	0.035972	0.000924	0.0010368
Female NHANES 2007–2008 mean intake, 50–59 years ratio	6.82 × 10^−1^	1.99 × 10^−1^	6.39 × 10^−2^	1.46 × 10^−2^	1.85 × 10^−2^	1.23 × 10^−2^	4.55 × 10^−3^	4.59 × 10^−3^	1.40 × 10^−4^	3.61 × 10^−5^	4.05 × 10^−5^
Female NHANES 2007–2008 mean intake, 50–59 years ratio with B12 set at 1	16,840	4907	1580	361	456	303	113	113	35	1	1
Male NHANES 2013–2014 mean intake (mg)	15.846	6.923	2.288	0.4731	0.5334	0.3145	0.12096	0.1107	0.0261	0.00078	0.0010602
Male NHANES 2013–2014 mean intake, 50–59 years ratio	5.95 × 10^−1^	2.60 × 10^−1^	8.59 × 10^−2^	1.78 × 10^−2^	2.00 × 10^−2^	1.18 × 10^−2^	4.54 × 10^−3^	4.16 × 10^−3^	9.80 × 10^−4^	2.93 × 10^−5^	3.98 × 10^−5^
Male NHANES 2013–2014 mean intake, 50–59 years ratio with B12 set at 1	14,946	6530	2158	446	503	297	114	104	25	1	1
Female NHANES 2013–2014 mean intake (mg)	18.525	5.616	2.15	0.414	0.4536	0.3336	0.11343	0.10488	0.048144	0.000902	0.000746
Female NHANES 2013–2014 mean intake, 50–59 years ratio	6.67 × 10^−1^	2.02 × 10^−1^	7.74 × 10^−2^	1.49 × 10^−2^	1.63 × 10^−2^	1.20 × 10^−2^	4.09 × 10^−3^	3.78 × 10^−3^	1.73 × 10^−4^	3.25 × 10^−5^	2.69 × 10^−5^
Female NHANES 2013–2014 mean intake, 50–59 years ratio with B12 set at 1	24,832	7528	2882	555	608	447	152	141	65	1	1
Dinner											
Male NHANES 2007–2008 mean intake (mg)	30.063	12.857	3.168	0.9	0.969	0.7068	0.198	0.21096	0.072504	0.002173	0.002329
Male NHANES 2007–2008 mean intake, 50–59 years ratio	6.12 × 10^−1^	2.62 × 10^−1^	6.45 × 10^−2^	1.83 × 10^−2^	1.97 × 10^−2^	1.44 × 10^−2^	4.03 × 10^−3^	4.29 × 10^−3^	1.48 × 10^−3^	4.42 × 10^−5^	4.74 × 10^−5^
Male NHANES 2007–2008 mean intake, 50–59 years ratio with B12 set at 1	12,906	5519	1360	386	416	303	85	91	31	1	1
Female NHANES 2007–2008 mean intake (mg)	29.682	8.48	2.574	0.6586	0.6698	0.5434	0.17192	0.1504	0.055016	0.001364	0.001469
Female NHANES 2007–2008 mean intake, 50–59 years ratio	6.90 × 10^−1^	1.97 × 10^−1^	5.99 × 10^−2^	1.53 × 10^−2^	1.56 × 10^−2^	1.26 × 10^−2^	4.00 × 10^−3^	3.50 × 10^−3^	1.28 × 10^−3^	3.17 × 10^−5^	3.42 × 10^−5^
Female NHANES 2007–2008 mean intake, 50–59 years ratio with B12 set at 1	20,208	5773	1752	448	456	370	117	102	37	1	1
Male NHANES 2013–2014 mean intake (mg)	30.858	12.341	3.744	0.9213	0.9906	0.7215	0.19488	0.2091	0.08091	0.001716	0.002065
Male NHANES 2013–2014 mean intake, 50–59 years ratio	6.16 × 10^−1^	2.46 × 10^−1^	7.48 × 10^−2^	1.84 × 10^−2^	1.98 × 10^−2^	1.44 × 10^−2^	3.89 × 10^−3^	4.18 × 10^−3^	1.62 × 10^−3^	3.43 × 10^−5^	4.12 × 10^−5^
Male NHANES 2013–2014 mean intake, 50–59 years ratio with B12 set at 1	14,946	5977	1813	446	480	349	94	101	39	1	1
Female NHANES 2013–2014 mean intake (mg)	25.194	8.856	3.182	0.72	0.6426	0.4726	0.16119	0.15504	0.065136	0.001066	0.00138
Female NHANES 2013–2014 mean intake, 50–59 years ratio	6.39 × 10^−1^	2.24 × 10^−1^	8.07 × 10^−2^	1.83 × 10^−2^	1.63 × 10^−2^	1.20 × 10^−2^	4.09 × 10^−3^	3.63 × 10^−3^	1.65 × 10^−3^	2.70 × 10^−5^	3.50 × 10^−5^
Female NHANES 2013–2014 mean intake, 50–59 years ratio with B12 set at 1	18,255	6417	2306	522	466	342	117	112	47	1	1
Snacks											
Male NHANES 2007–2008 mean intake (mg)	23.686	4.186	1.848	0.315	0.357	0.279	0.1452	0.0879	0.007632	0.000636	0.000674
Male NHANES 2007–2008 mean intake, 50–59 years ratio	7.66 × 10^−1^	1.35 × 10^−1^	5.98 × 10^−2^	1.02 × 10^−2^	1.15 × 10^−2^	9.03 × 10^−3^	4.70 × 10^−3^	2.84 × 10^−3^	2.47 × 10^−4^	2.06 × 10^−5^	2.18 × 10^−5^
Male NHANES 2007–2008 mean intake, 50–59 years ratio with B12 set at 1	35,127	6208	2741	467	529	414	215	130	11	1	1
Female NHANES 2007–2008 mean intake (mg)	23.571	3.604	2.262	0.3382	0.3546	0.2431	0.14736	0.0752	0.009522	0.000748	0.000605
Female NHANES 2007–2008 mean intake, 50–59 years ratio	7.70 × 10^−1^	1.18 × 10^−1^	7.39 × 10^−2^	1.00 × 10^−2^	1.16 × 10^−2^	7.94 × 10^−3^	4.82 × 10^−3^	2.46 × 10^−3^	3.11 × 10^−4^	2.44 × 10^−5^	1.98 × 10^−5^
Female NHANES 2007–2008 mean intake, 50–59 years ratio with B12 set at 1	38,973	5959	3740	559	586	402	244	124	16	1	1
Male NHANES 2013–2014 mean intake (mg)	17.514	5.418	2.288	0.4731	0.4064	0.296	0.16128	0.0984	0.014355	0.000624	0.000725
Male NHANES 2013–2014 mean intake, 50–59 years ratio	6.57 × 10^−1^	2.03 × 10^−1^	8.58 × 10^−2^	1.77 × 10^−2^	1.52 × 10^−2^	1.11 × 10^−2^	6.05 × 10^−3^	3.69 × 10^−3^	5.38 × 10^−4^	2.34 × 10^−5^	2.72 × 10^−5^
Male NHANES 2013–2014 mean intake, 50–59 years ratio with B12 set at 1	24,144	7469	3154	652	560	408	222	136	20	1	1
Female NHANES 2013–2014 mean intake (mg)	14.82	3.024	1.634	0.252	0.3591	0.1946	0.13134	0.07296	0.012744	0.000533	0.000485
Female NHANES 2013–2014 mean intake, 50–59 years ratio	7.23 × 10^−1^	1.47 × 10^−1^	7.97 × 10^−2^	1.23 × 10^−2^	1.75 × 10^−2^	9.49 × 10^−3^	6.41 × 10^−3^	3.56 × 10^−3^	6.22 × 10^−4^	2.60 × 10^−5^	2.37 × 10^−5^
Female NHANES 2013–2014 mean intake, 50–59 years ratio with B12 set at 1	30,563	6236	3370	520	741	401	271	150	26	1	1

Ratio values are rounded. Abbreviations: vitamin A (as retinol activity equivalents (RAE)), thiamin (B_1_), riboflavin (B_2_), niacin (B_3_), pyridoxine (B_6_), cobalamin (B_12_), folate (as dietary folate equivalent (DFE)), vitamin C (C), vitamin D (D), vitamin E (E) and vitamin K (Vit K).

**Table 5:** Minerals and vitamins combined from Dietary Reference Intakes (DRI) for males and females 51–70 years and mean intakes from food derived from National Health and Nutrition Examination Survey (NHANES) 2013–2014 data for males aged 51–59 years, their ratios and ratios relative to vitamin B_12_.

Nutrient	Male						Female					
	DRI (mg)	DRI Ratio	DRI Ratio with Vit B_12_ Set at 1	NHANES Intake (mg)	NHANES Intake Ratio	NHANES Ratio with Vit B_12_ Set at 1	DRI (mg)	DRI Ratio	DRI Ratio with Vit B_12_ Set at 1	NHANES Intake (mg)	NHANES Intake Ratio	NHANES Ratio with Vit B_12_ Set at 1
**K**	4700	0.56855349	1,958,333	3110	0.30165623	557,348	4700	0.56315331	1,958,333	2390	0.31035013	640,751
**Na**	1300	0.15725948	541,667	4074	0.39515997	730,108	1300	0.15576581	541,667	2894	0.37579635	775,871
**Ca**	1000	0.12096883	416,667	1589	0.15412597	284,767	1200	0.14378382	500,000	1177	0.15283770	315,550
**P**	700	0.08467818	291,667	1013	0.09825652	181,541	700	0.08387390	291,667	828	0.10751879	221,984
**Mg**	420	0.05080691	175,000	360	0.03491841	64,516	320	0.03834235	133,333	278	0.03609930	74,531
**Vit C**	90	0.01088719	37,500	83.4	0.00808943	14,946	75	0.00898649	31,250	74.1	0.00962215	19,866
**B_3_**	16	0.00193550	6667	30.1	0.00291957	5394	14	0.00167748	5833	21.6	0.00280484	5791
**Vit E**	15	0.00181453	6250	10.4	0.00100875	1864	15	0.00179730	6250	8.6	0.00111674	2306
**Zn**	11	0.00133066	4583	17	0.00164892	3047	8	0.00095856	3333	13.1	0.00170108	3512
**Fe**	8	0.00096775	3333	12.9	0.00125124	2312	8	0.00095856	3333	9.1	0.00118167	2440
**B_6_**	1.7	0.00020565	708	2.49	0.00024152	446	1.5	0.00017973	625	1.8	0.00023374	483
**B_2_**	1.3	0.00015726	542	2.54	0.00024637	455	1.1	0.00013180	458	1.89	0.00024542	507
**B_1_**	1.2	0.00014516	500	1.85	0.00017944	332	1.1	0.00013180	458	1.39	0.00018050	373
**RAE**	0.9	0.00010887	375	0.672	0.00006518	120	0.7	0.00008387	292	0.597	0.00007752	160
**Cu**	0.9	0.00010887	375	1.5	0.00014549	269	0.9	0.00010784	375	1.1	0.00014284	295
**DFE**	0.4	0.00004839	167	0.615	0.00005965	110	0.4	0.00004793	167	0.456	0.00005921	122
**Vit K**	0.12	0.00001452	50	0.1305	0.00001266	23	0.09	0.00001078	38	0.1416	0.00001839	38
**Se**	0.055	0.00000665	23	0.1405	0.00001363	25	0.055	0.00000659	23	0.0971	0.00001261	26
**D**	0.015	0.00000181	6	0.0052	0.00000050	1	0.015	0.00000180	6	0.0041	0.00000053	1
**B_12_**	0.0024	0.00000029	1	0.00558	0.00000054	1	0.0024	0.00000029	1	0.00373	0.00000048	1
**TOTAL**	8266.5924	1		10,309.74878	1		8345.8624	1		7700.97953	1	

**Table 6:** Tolerable Upper Intake Levels (ULs) and Dietary Reference Intakes (DRIs) for males 51–70 years, their ratios and ratios relative to vitamin D and a hypothetical diet containing the DRI levels but with vitamin C at the UL and both ratios.

Nutrient	UL			DRI			DRI with Vit C at UL		
	mg	Ratio	Ratio with Vit D Set at 1	mg	Ratio	Ratio with Vit D Set at 1	mg	Ratio	Ratio with Vit D Set at 1
**P**	4000	0.34678573	40,000	700	0.22264843	46,667	700	0.13850498	46,667
**Na**	2300	0.19940179	23,000	1300	0.41348995	100,000	1300	0.25722353	86,667
**Ca**	2000	0.17339286	20,000	1000	0.31806919	66,667	1000	0.19786425	66,667
**Vit C**	2000	0.17339286	20,000	90	0.02862623	6000	2000	0.39572851	133,333
**Vit E**	1000	0.08669643	10,000	15	0.00477104	1000	15	0.00296796	1000
**B_6_**	100	0.00866964	1000	1.7	0.00054072	113	1.7	0.00033637	113
**Fe**	45	0.00390134	450	8	0.00254455	533	8	0.00158291	533
**Zn**	40	0.00346786	400	11	0.00349876	733	11	0.00217651	733
**B_3_**	35	0.00303438	350	16	0.00508911	1067	16	0.00316583	1067
**Cu**	10	0.00086696	100	0.9	0.00028626	60	0.9	0.00017808	60
**RAE**	3	0.00026009	30	0.9	0.00028626	60	0.9	0.00017808	60
**DFE**	1	0.00008670	10	0.4	0.00012723	27	0.4	0.00007915	27
**Se**	0.4	0.00003468	4	0.055	0.00001749	4	0.055	0.00001088	4
**Vit D**	0.1	0.00000867	1	0.015	0.00000477	1	0.015	0.00000297	1
**TOTAL**	11,534.5	1		3143.97	1		5053.97	1	

**Table 7:** Micronutrient Dietary Reference Intakes (DRI) for males and females 51–70 years, the percentage contribution of each micronutrient to the total, the intakes from National Health and Nutrition Examination Survey (NHANES) 2013–2014 data, for males and females, from food, aged 51–59 years and the projected intake, per DRI proportions, based on the total intake.

Nutrient	Male				Female			
	DRI 51–70 Years (mg)	Percent of Total DRI (%)	2013–2014 NHANES Intake—Age 50–59 (mg)	Hypothetical Intake in Proportion to DRI (mg)	DRI 51–70 Years (mg)	Percent of Total DRI (%)	2013–2014 NHANES Intake—Age 50–59 (mg)	Hypothetical Intake in Proportion to DRI (mg)
**K**	4700	56.85535	3110	5861.64370	4700	56.31533	2390	4336.83208
**Na**	1300	15.72595	4074	1621.30570	1300	15.57658	2894	1199.54930
**Ca**	1000	12.09688	1589	1247.15823	1200	14.37838	1177	1107.27628
**P**	700	8.46782	1013	873.01076	700	8.38739	828	645.91116
**Mg**	420	5.08069	360	523.80646	320	3.83424	278	295.27367
**Vit C**	90	1.08872	83.4	112.24424	75	0.89865	74.1	69.20477
**B_3_**	16	0.19355	30.1	19.95453	14	0.16775	21.6	12.91822
**Vit E**	15	0.18145	10.4	18.70737	15	0.17973	8.6	13.84095
**Zn**	11	0.13307	17	13.71874	8	0.09586	13.1	7.38184
**Fe**	8	0.09678	12.9	9.97727	8	0.09586	9.1	7.38184
**B_6_**	1.7	0.02056	2.49	2.12017	1.5	0.01797	1.8	1.38410
**B_2_**	1.3	0.01573	2.54	1.62131	1.1	0.01318	1.89	1.01500
**B_1_**	1.2	0.01452	1.85	1.49659	1.1	0.01318	1.39	1.01500
**RAE**	0.9	0.01089	0.672	1.12244	0.7	0.00839	0.597	0.64591
**Cu**	0.9	0.01089	1.5	1.12244	0.9	0.01078	1.1	0.83046
**DFE**	0.4	0.00484	0.615	0.49886	0.4	0.00479	0.456	0.36909
**Vit K**	0.12	0.00145	0.1305	0.14966	0.09	0.00108	0.1416	0.08305
**Se**	0.055	0.00067	0.1405	0.06859	0.055	0.00066	0.0971	0.05075
**D**	0.015	0.00018	0.0052	0.01871	0.015	0.00018	0.0041	0.01384
**B_12_**	0.0024	0.00003	0.00558	0.00299	0.0024	0.00003	0.00373	0.00221
**TOTAL**	8266.5924	100	10,309.74878		8345.8624	100	7700.97953	

## Supplementary Materials

The following are available online at www.mdpi.com/2072-6643/10/1/107/s1, Figure S1: Dietary Reference Intakes (DRI), Daily Values (DV) and mean daily mineral intakes from food for males age 50 to 59 for the 2007–2008 and 2013–2014 National Health and Nutrition Examination Survey (NHANES) derived data, Figure S2: Dietary Reference Intakes (DRI), Daily Values (DV) and mean daily mineral intakes from food for females age 50 to 59 for the 2007–2008 and 2013–2014 National Health and Nutrition Examination Survey (NHANES) derived data, Figure S3: Dietary Reference Intakes (DRI), Daily Values (DV) and mean daily vitamin intakes from food for males age 50 to 59 for the 2007–2008 and 2013–2014 National Health and Nutrition Examination Survey (NHANES) derived data, Figure S4: Dietary Reference Intakes (DRI), Daily Values (DV) and mean daily vitamin intakes from food for females age 50 to 59 for the 2007–2008 and 2013–2014 National Health and Nutrition Examination Survey (NHANES) derived data.
