# Supplementary material for: Utilizing Dietary Micronutrient Ratios in Nutritional Research May be More Informative than Focusing on Single Nutrients
Source: Nutrients. 2018 Jan 19;10(1):107. doi: 10.3390/nu10010107 (PMC5793335; doi:10.3390/nu10010107)
Supplement: Supplementary file 1 [file nutrients-10-00107-s001.pdf]

Concept Paper

Utilizing Dietary Micronutrient Ratios in Nutritional Research May be More Informative than Focusing on Single Nutrients

Owen J. Kelly [1,*], Jennifer C. Gilman [1] and Jasminka Z. Ilich [2]

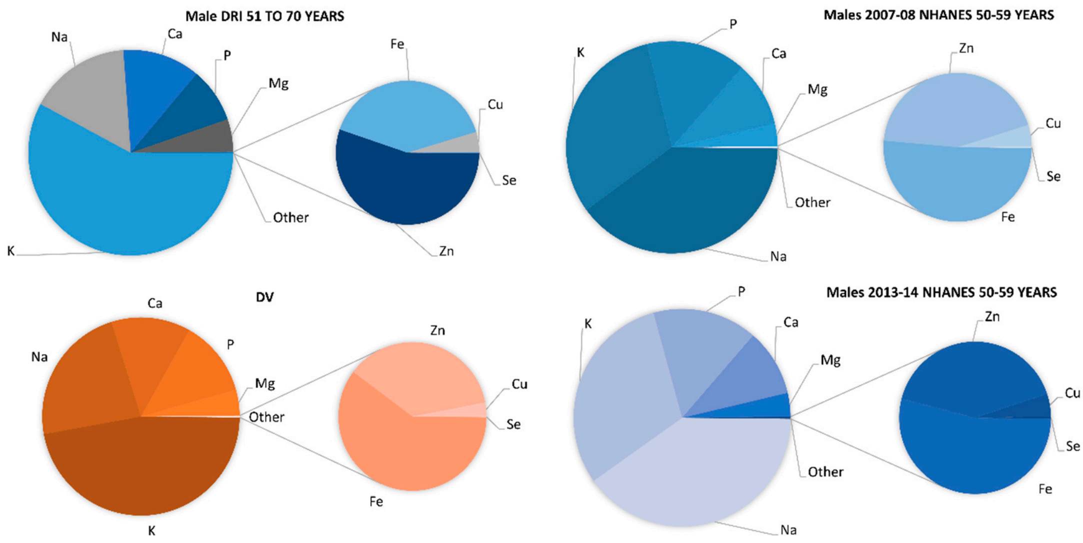

Figure S1. Dietary Reference Intakes (DRI), Daily Values (DV) and mean daily mineral intakes from food for males age 50 to 59 for the 2007–2008 and 2013–2014 National Health and Nutrition Examination Survey (NHANES) derived data.

Data are expressed as the percent of total intake. The secondary pie chart contains the lowest values. Abbreviations: calcium (Ca), phosphorus (P), magnesium (Mg), iron (Fe), zinc (Zn), copper (Cu), selenium (Se), potassium (K) and sodium (Na).

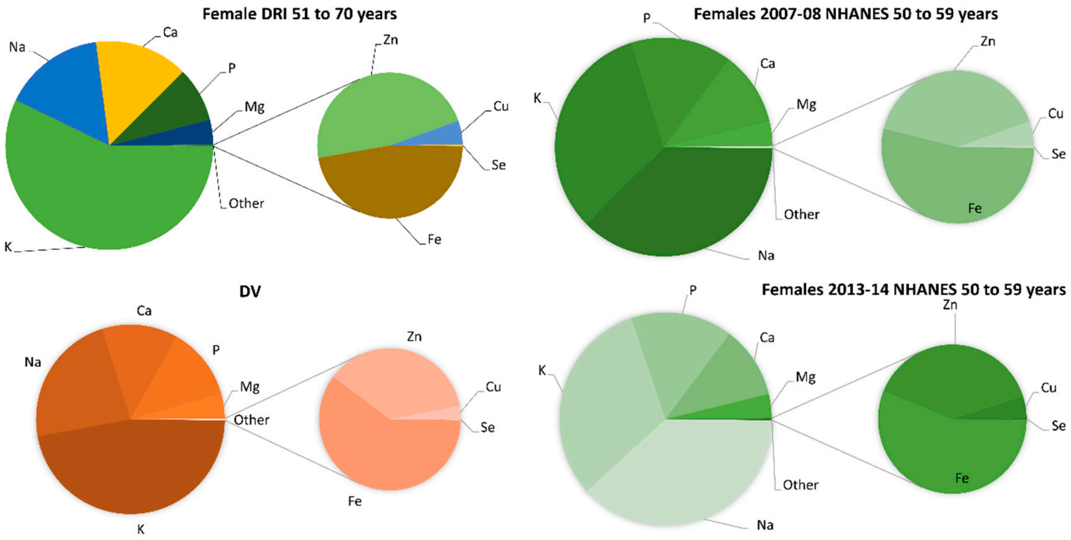

Figure S2. Dietary Reference Intakes (DRI), Daily Values (DV) and mean daily mineral intakes from food for females age 50 to 59 for the 2007–2008 and 2013–2014 National Health and Nutrition Examination Survey (NHANES) derived data.

Data are expressed as the percent of total intake. The secondary pie chart contains the lowest values. Abbreviations: calcium (Ca), phosphorus (P), magnesium (Mg), iron (Fe), zinc (Zn), copper (Cu), selenium (Se), potassium (K) and sodium (Na).

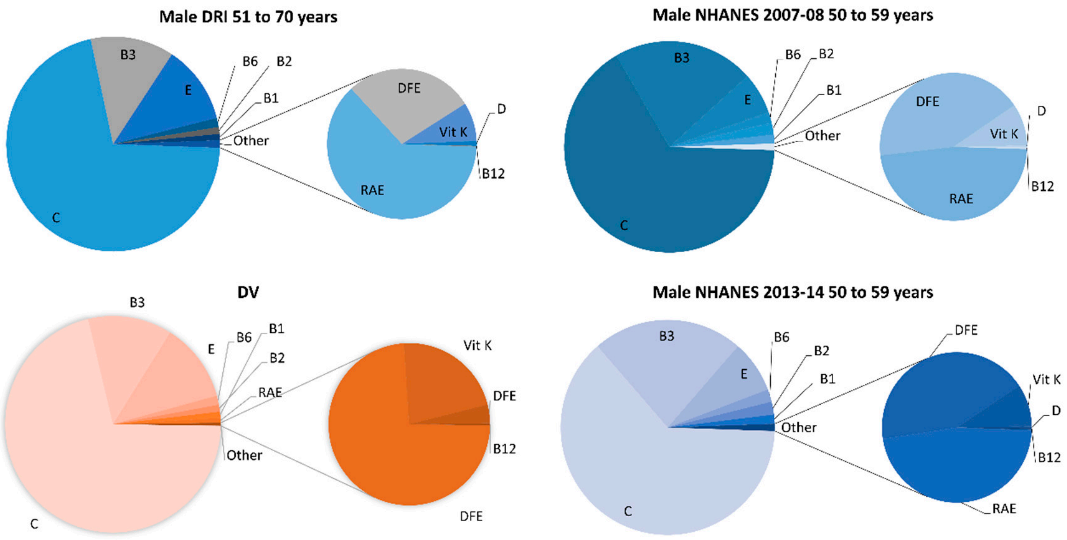

Figure S3. Dietary Reference Intakes (DRI), Daily Values (DV) and mean daily vitamin intakes from food for males age 50 to 59 for the 2007–2008 and 2013–2014 National Health and Nutrition Examination Survey (NHANES) derived data.

Data are expressed as the percent of total intake. The secondary pie chart contains the lowest values. Vitamins include A (as retinol activity equivalents (RAE)), thiamin (B1), riboflavin (B2), niacin (B3), pyridoxine (B6), cobalamin (B12), folate (as dietary folate equivalent (DFE)), vitamin C (C), vitamin D (D), vitamin E (E) and vitamin K (Vit K).

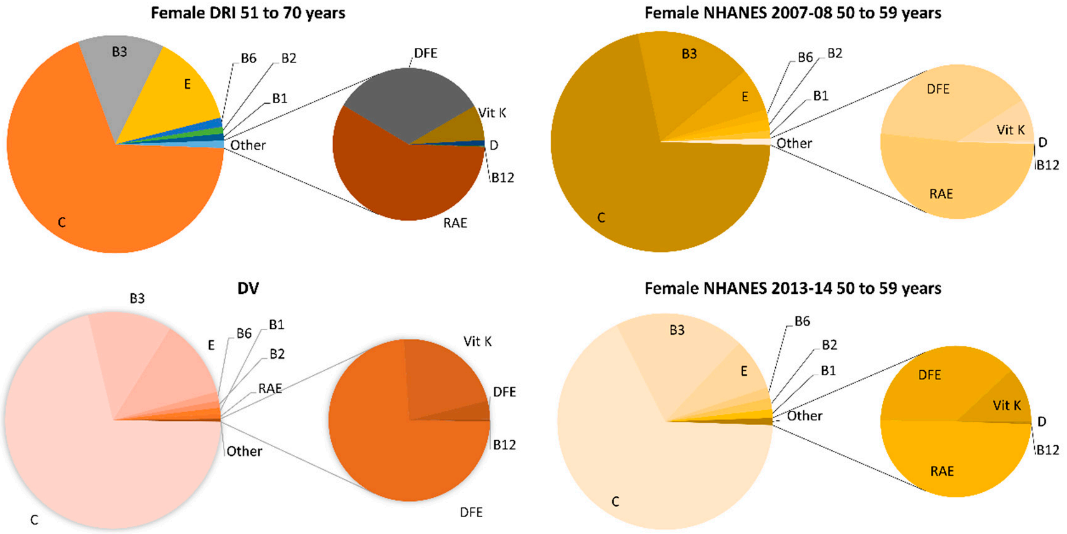

Figure S4. Dietary Reference Intakes (DRI), Daily Values (DV) and mean daily vitamin intakes from food for females age 50 to 59 for the 2007–2008 and 2013–2014 National Health and Nutrition Examination Survey (NHANES) derived data.

Data are expressed as the percent of total intake. The secondary pie chart contains the lowest values. Vitamins include A (as retinol activity equivalents (RAE)), thiamin (B1), riboflavin (B2), niacin (B3), pyridoxine (B6), cobalamin (B12), folate (as dietary folate equivalent (DFE)), vitamin C (C), vitamin D (D), vitamin E (E) and vitamin K (Vit K).
